# New Books

**Published:** 2006-05

Biochemical Mechanisms of Detoxification in Higher Plants: Basis of Phytoremediation

G. Kvesitadze, G. Khatisashvili, T.Sadunishvili, J.J. Ramsden

New York:Springer, 2006. 256 pp. ISBN: 3-540-28996-8, $169

Building Healthy Communities in Environmental Justice Areas

Janine M. Legg

North Charleston, SC:BookSurge Publishing, 2006. 290 pp. ISBN: 1-4196-2757-0, $23.99

Clinical Applications of PCR

Y. M. Dennis Lo, Rossa W.K. Chiu, K.C. Allen Chan

Totowa, NJ:Humana Press, 2006. 224 pp. ISBN: 1-58829-348-3, $99.50

Ecological Informatics: Scope, Techniques and Applications

Friedrich Recknagel, ed.

New York:Springer, 2006. 496 pp. ISBN: 3-540-28383-8, $139

Energy in the 21st Century

John R Fanchi

Hackensack, NJ:World Scientific Publishing Co., 2006. 256 pp. ISBN: 981-256-185-4, $52

Environmental Impacts of Treated Wood

Timothy G. Townsend, Helena Solo-Gabriele

Boca Raton, FL:CRC Press, 2006. 520 pp. ISBN: 0-8493-6495-7, $139.95

Environmental Risk Assessment

Ian Lerche, Walter Glaesser,

New York:Springer, 2006. 343 pp. ISBN: 3-540-26249-0, $139

Fluoride in Drinking Water: A Scientific Review of EPA’s Standards

Committee on Fluoride in Drinking Water, National Research Council

Washington, DC:National Academies Press, 2006. 576 pp. ISBN: 0-309-10129-8, $63

Hazardous Substances and Human Health

T.M. Bachmann

Burlington, MA:Elsevier, 2006. 612 pp. ISBN: 0-444-52218-2, $135

Institutional Interaction in Global Environmental Governance: Synergy and Conflict among International and EU Policies

Sebastian Oberthür, Thomas Gehring, eds.

Cambridge, MA:MIT Press, 2006. 424 pp. ISBN: 0-262-05115-X, $68

Medical Treatment of Intoxications and Decontamination of Chemical Agents in the Area of Terrorist Attack

Christophor Dishovsky, Alexander Pivovarov, Hendrik Benschop, eds.

New York:Springer, 2006. 233 pp. ISBN: 1-4020-4169-1, $64.95

Nanotechnology for Environmental Remediation

Sung Hee Joo, I. Francis Cheng

New York:Springer, 2006. 166 pp. ISBN: 0-387-28825-2, $89.95

New and Emerging Proteomic Techniques

Dobrin Nedelkov, Randall W. Nelson

Totowa, NJ:Humana Press, 2006. 260 pp. ISBN: 1-58829-519-2, $99.50

Perchlorate: Environmental Occurrence, Interactions and Treatment

Baohua Gu, John D. Coates, eds.

New York:Springer, 2006. 412 pp. ISBN: 0-387-31114-9, $99

Persistent Organic Pollutants in the Great Lakes

Ronald Hites, ed.

New York:Springer, 2006. 441 pp. ISBN: 3-540-29168-7, $309

Proteomics for Biological Discovery

Timothy D. Veenstra, John R. Yates

Hoboken, NJ:John Wiley & Sons, 2006. 332 pp. ISBN: 0-471-16005-9, $69.95

The Environment in Asia Pacific Harbours

Eric Wolanski, ed.

New York:Springer, 2006. 497 pp. ISBN: 1-4020-3654-X, $209

Toxicity Testing for Assessment of Environmental Agents: Interim Report

Committee on Toxicity Testing and Assessment of Environmental Agents, National Research Council

Washington, DC:National Academies Press, 2006. 270 pp. ISBN: 0-309-10093-3, $39

Uncertainty Underground: Yucca Mountain and the Nation’s High-Level Nuclear Waste

Allison M. Macfarlane, Rodney C. Ewing, eds.

Cambridge, MA:MIT Press, 2006. 416 pp. ISBN: 0-262-13462-4, $72

What’s the Beef?: The Contested Governance of European Food Safety

Christopher Ansell, David Vogel, Eds.

Cambridge, MA:MIT Press, 2006. 400 pp. ISBN: 0-262-01225-1, $67

